# Lineage diversification, homo- and heterologous reassortment and recombination shape the evolution of chicken orthoreoviruses

**DOI:** 10.1038/srep36960

**Published:** 2016-11-10

**Authors:** Szilvia L. Farkas, Szilvia Marton, Eszter Dandár, Renáta Kugler, Bence Gál, Ferenc Jakab, Ádám Bálint, Sándor Kecskeméti, Krisztián Bányai

**Affiliations:** 1 Institute for Veterinary Medical Research, Centre for Agricultural Research, Hungarian Academy of Sciences, Hungária krt. 21, Budapest 1143, Hungary; 2 United Szent István és Szent László Hospital − Clinic, Nagyvárad tér 1, Budapest 1097, Hungary; 3 János Szentágothai Research Centre, University of Pécs, Ifjúság útja 20, Pécs 7624, Hungary; 4 Veterinary Diagnostic Directorate, National Food Chain Safety Office, Tábornok u. 2, Budapest 1143, Hungary; 5 Veterinary Diagnostic Directorate, National Food Chain Safety Office, Bornemissza u. 3-7, Debrecen 4031, Hungary

## Abstract

The near complete genome sequences of ten field avian orthoreovirus (ARV) strains collected from young chicken between 2002 and 2011 in Hungary have been determined in order to explore the genetic diversity and evolutionary mechanisms affecting ARVs in this region. Sequence analyses and phylogenetic calculations revealed similar geographic distribution and distinct evolution in case of eight studied strains that were closely related to the recently described Hungarian strain T1781. The remaining two strains showed the highest similarity with the US origin AVS-B. The topology of the phylogenetic trees of certain segments was affected by several potential homologous reassortment events between strains of Hungarian, Chinese and US origin. Analyzing the μB gene a possible heterologous reassortment event was identified in three Hungarian strains. Recombination events were detected in as much as a dozen cases implying that beside point mutations and reassorment this mechanism also plays an important role in the diversification of ARVs. All these mechanisms in concert may explain the reduced effectiveness of immunization using commercial vaccine strains.

Avian orthoreoviruses (ARVs; family *Reoviridae*, subfamily *Spinareovirinae*, genus *Orthoreovirus*) are non-enveloped, icosahedral particles with a diameter of 70–80 nm^1^. All currently known reovirus strains from chicken, turkey, partridge and waterfowl species belong to the species *Avian orthoreovirus*, while other ARV-like strains, including the Tvärminne avian orthoreovirus (TVAV), the Bulbul orthoreovirus (Pycno-1) and the closely related Steller sea lion reovirus (SSRV), and the Psittacine reovirus strain Ge01, were suggested to be prototype members of further novel species, respectively^2^,^3^,^4^.

Like other members of the genus, ARVs possess a double-stranded RNA genome consisting of 10 segments enclosed within two capsid protein layers. The segments can be separated by gel electrophoresis based on their different size, namely large (L), medium (M), and small (S)^5^, which can further be subdivided into 3 L (L1−L3), 3 M (M1−M3) and 4 S (S1−S4), respectively. With the exception of the S1 or S4 segment which might contain two or three open reading frames (depending on strain), all genomic segments encode a single protein, enabling the virus to express at least eight primary structural and three or four non-structural (μNS, σNS, p10 and p17) viral proteins. While the inner core is made up of five highly conserved proteins (λA, λB, λC, μA, and σA), the outer capsid consists of three more variable proteins (μB, σB, and σC).

ARV infections are widespread and had been associated with various clinical manifestations in domestic poultry^6^. In chicken, viral arthritis/tenosynovitis with the inflammation of the tibiotarsal-tarsometatarsal joints and rupture of the gastrocnemius tendon is the most commonly diagnosed form in 4–16 weeks old broiler chickens. Runting-stunting syndrome is more common in younger, 2–3-week-old chickens, and characterised by slower development, uneven growth rate, bone formation disorder and abnormal feathering (“helicopter disease”). Reovirus infections were also diagnosed in connection with hepatitis, myocarditis, central nervous system infections, and immuno-suppression of chickens^7^,^8^,^9^,^10^.

Although the number of complete genome sequences available in GenBank is still limited, sequence data collected in the last few years suggest that the genetic material of ARV strains circulating in domestic poultry is continuously changing due to the well-known evolutionary mechanisms of reoviruses, resulting in genetically and antigenically heterogeneous strains. As observed in other RNA viruses, point mutation which is the primary mechanism of genetic and antigenic drift is relatively common. Antigenic shift, another evolutionary strategy, occurs when cognate genome segments reassort, and this mechanism is thought to be the driving force of the emergence of novel strains causing disease in poultry^1^. As a consequence of diverse evolutionary mechanisms, the commercially available ARV vaccines are not able to provide adequate protection against newly emerging field strains leading to outbreaks of disease in vaccinated poultry flocks^11^,^12^,^13^.

In this study we determined the near complete genome sequences of ten ARV field strains collected from young chicken with different clinical signs over a 10-year period in Hungary (Table 1) in order to gain more genetic data about the circulating strains, and to determine their relatedness to contemporary, predominantly Asian and US origin ARV strains, the genomic sequences of which are accessible in GenBank. Identifying intra-segmental recombination and heterologous reassortment events permits new insight into the evolutionary mechanisms of ARV strains commonly detected in chicken. European ARV strains have been underrepresented in previous studies thus this survey provides important new information about the geographic distribution and evolutionary origin of chicken ARVs.

## Results and Discussion

### Genomic organization and coding potential

The near complete genome sequences of ten, randomly selected Hungarian chicken ARV isolates had been determined by a modified sequence-independent, single-primer amplification method coupled with next-generation sequencing. The genomic organization of these ARV strains was similar and corresponded with that of the previously described reference ARV strains, AVS-B and S1133. With the exception of the S1, all genomic segments were found to encode a single protein and the homologues of the following orthoreoviral proteins were identified by open reading frame prediction and sequence comparison: λA, λB, λC, μA, μB, μNS, σA, σB, σC, σNS, p10 and p17. Where available, the 5′ and 3′ terminal sequences (GCUUUU[U/C] and UCAUC) were conserved among all segments as observed in other ARVs described so far^5^,^14^.

### Sequence homology and phylogenetic relationship among strains

**Figure 1:**
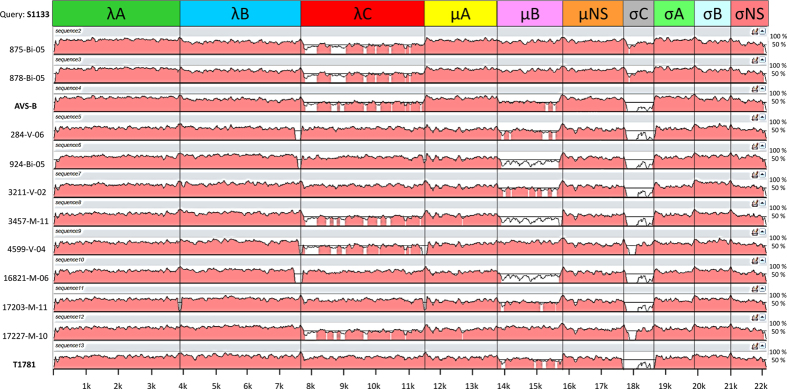
Results of the mVISTA analysis of the concatenated genome sequences of the ten studied Hungarian avian orthoreovirus strains and the recently described T1781, and the US origin reference strains, S1133 and AVS-B. Strain S1133 was used as query.

Sequence comparison of the Hungarian strains revealed 71.1–100/83.0–100% nucleotide sequence identity and aa sequence similarity values with the exception of the μB and σC genes where higher genetic variability was observed, and these values ranged between 62.7–99.8% nt and 71.3–99.7% aa for μB and 52.9–100% nt and 46.2–100% aa for σC, respectively (Fig. 1, Table 2).

Eight out of ten Hungarian strains (284-V-06, 924-Bi-05, 3211-V-02, 3457-M-11, 4599-V-04, 16821-M-06, 17203-M-06, 17227-M-10) showed the highest homology with T1781, another Hungarian ARV strain we characterized recently^8^. The common evolutionary origin of these strains is clearly reflected by the results of the analysis performed with the nt and aa sequences of the λA gene. When comparing these strains with T1781, the nt identity and aa similarity values were high (falling between 91.1–95.1% and 98.8–99.3%, respectively). Lower values were obtained in the calculations performed with the sequences of US origin reference strains, S1133 and AVS-B, isolated in 1971 and 2006, respectively. In the case of these strains the nt identity values ranged between 83.1–84.1% and the aa similarity values were between 82.5–83.3% and 97–97.6%, respectively. Sequence analyses revealed that the remaining two strains 875-Bi-05 and 878-Bi-05 were most closely related to each other showing high, 98.8–100% nt sequence identity and 99.7–100% aa sequence similarity, and with the exception of the σC (54.8% nt and 50.5% aa identities) and μB genes (sequence identity ranges, 76.6% nt, 89–89.3% aa), these strains showed the highest similarity with the US strain, AVS-B (sequence identity ranges, 89.1–98.8% nt, 83.8–100% aa).

**Figure 2:**
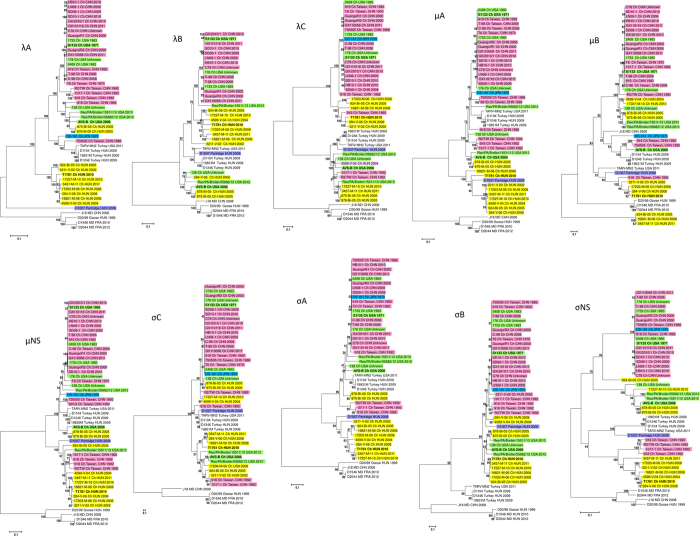
Unrooted nucleotide sequence based phylogenetic trees showing the clustering of avian orthoreoviruses based on the λA, λB, λC, μA, μB, μNS, σC, σA, σB and σNS protein coding genes of viruses available from GenBank. Phylogenetic calculations were carried out using the maximum-likelihood method applying the best-fit models calculated for each gene. The scale bar is proportional to the genetic distance.

Phylogenetic analyses of the 10 genomic segments resulted in different tree topologies suggesting that certain segments may have been constituted by distinct evolutionary mechanisms; several hypothetical reassortment events between strains originating from different geographic regions were observed indicating that this phenomenon is relatively common in the *Avian orthoreovirus* species (Fig. 2). With the exception of the μB gene in all phylogenies host specific evolution of gallinaceous and waterfowl origin ARVs was evident^15^. The geographic origin of different ARV strains based on their genetic relatedness could also be observed especially in the case of the more conserved core proteins. Our calculations suggested that numerous reassortment events occurred recently between Hungarian, US, and Asian strains affecting mainly the outer capsid and non-structural protein coding genes, therefore the geographic origin of strains in these particular cases was less apparent. Phylogenetic analysis of the μB and σC outer capsid proteins, responsible for virus entry and transcriptase activation, and antigenic properties, respectively, revealed greater genetic distances between the different ARV strains. In detail, most of the studied Hungarian strains (284-V-06, 924-Bi-05, 3211-V-02, 3457-M-11, 4599-V-04, 16821-M-06, 17203-M-06, 17227-M-10) formed a separate cluster together and with T1781, particularly in the λA, λB, μA, μNS, σA, σB and σNS gene phylogenies, suggesting that these strains evolved into evolutionary lineages distinct from those detected on other continents, mainly Asia and the Americas. In the λC, μB, μNS, σB, σC, σNS phylogenies some reassortant US (only distantly related to S1133) and/or Chinese origin strains clustered together with the Hungarian strains. In contrast, strains 875-Bi-05 and 878-Bi-05 were most closely related to each other and US origin strains, AVS-B, 138, Reo/PA/Broiler/05682/12 and -15511/13. With the exception of the μB, σA and σC genes, 875-Bi-05 and 878-Bi-05 clustered together with AVS-B. Chinese strains, 916, 918, R2/TW and 1017-1 collected in 1992 in Taiwan, appeared together in the μNS, σB and σNS phylogenies, but some of the strains formed common lineages with the Hungarian isolates in the λC, μB, σC, and σNS calculations. None of the Hungarian strains formed common clusters with the usual vaccine strains, S1133, 1733 and 2408, respectively, clearly indicating that the studied Hungarian strains were not vaccine strain origin ARVs.

### Detection of a putative heterologous reassortment event

**Figure 3:**
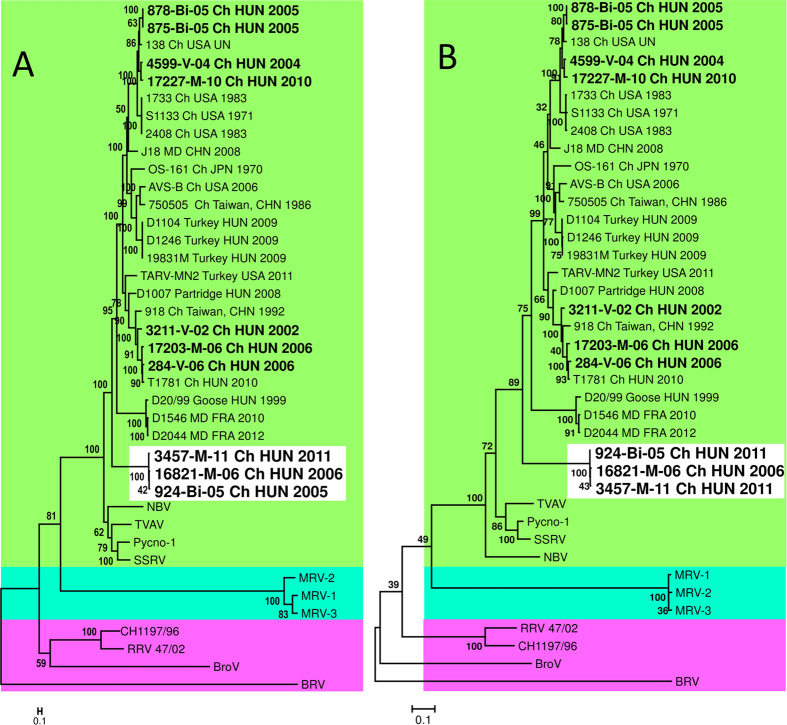
Phylogenetic analysis of the nucleotide (**A**) and amino acid (**B**) sequences of the μB gene of orthoreoviruses.

Interestingly, when analyzing the μB gene, three Hungarian strains, 924-Bi-05, 3457-M-11, and 16821-M-06, formed a monophyletic group distinct from both waterfowl and other gallinaceous ARVs, suggesting that these strains might have acquired this gene through a reassortment event from a different, yet unknown host species (Figs 2 and 3). Moreover, based on sequence comparison it cannot be excluded that even an inter-orthoreovirus-species reassortment might have occurred in this case given that nt identity values between 924-Bi-05, 3457-M-11 and 16821-M-06 and the other ARVs (63.9–66.2%) were similar to the values when these strains were compared with ARV-like viruses including Nelson Bay Virus (NBV), TVAV, Pycno-1 and SSRV (59.6–64.6%) (Table 3). Calculated nt identity values between the NBV and TVAV, Pycno-1 and SSRV, not classified into the same orthoreovirus species, were even higher 65.5–66.1%. At the aa level the similarity values calculated with S1133, AVS-B and T1781 ranged between 71.7–73.4%, while these were lower, 64.4–69.1%, when calculations were performed with the ARV-like viruses (Table 3), suggesting that some sort of adaptation process to the gallinaceous host and/or fine-tuning among interacting virion components of true chicken origin ARVs has already begun. In some positions these strains had a unique aa composition characteristic only to these three Hungarian strains when compared with other known members of the genus *Orthoreovirus* (data not shown). These Hungarian reassortant strains were collected in 2005, 2006 and 2011 in Hungary and strains possessing a closely related gene have never been detected before and after in any other countries. The first seven nt of the 5′ terminal sequences (GCUUUUU) of μB of the three strains showed high level of conservation with ARVs, implicating that this conserved terminal sequence may have been a prerequisite of the acquisition of this gene by reassortment. Apart from this conserved motif the 5′ untranslated region (UTR) sequences showed considerable variability and were 4 nt shorter (25 nt) when compared with the μB encoding genome segment of other ARV strains supporting the theory of the heterologous reassortment. However, alignment of the 5′ UTR containing orthoreoviruses belonging to other genera and unclassified strains revealed the highest similarity with ARV-like viruses, especially with NBV and Pulau virus (data not shown).

Three distinct lineages of orthoreoviruses have been described in previous studies, suggesting a common evolutionary history of each of the following clades: (i) the reptilian orthoreovirus strains with Baboon orthoreovirus and Broome virus, (ii) the classical mammalian orthoreoviruses, (iii) the avian orthoreoviruses along with NBV, TVAV and SSRV. It is possible that reassortment might occur between species belonging to the monophyletic group of the third clade, but the exact genetic breakpoint remains to be discovered. The genetic and phylogenetic distance is considerably smaller between representative members of the different species in the ARV-like clade, than that of the two other clades. Although it is documented that reassortment occurs only between members of a given orthoreovirus species^1^, to the best of our knowledge co-infection studies have not been systemically performed to rule out the possibility of an inter-reovirus-species reassortment event.

### Intrasegmental recombination events

**Figure 4:**
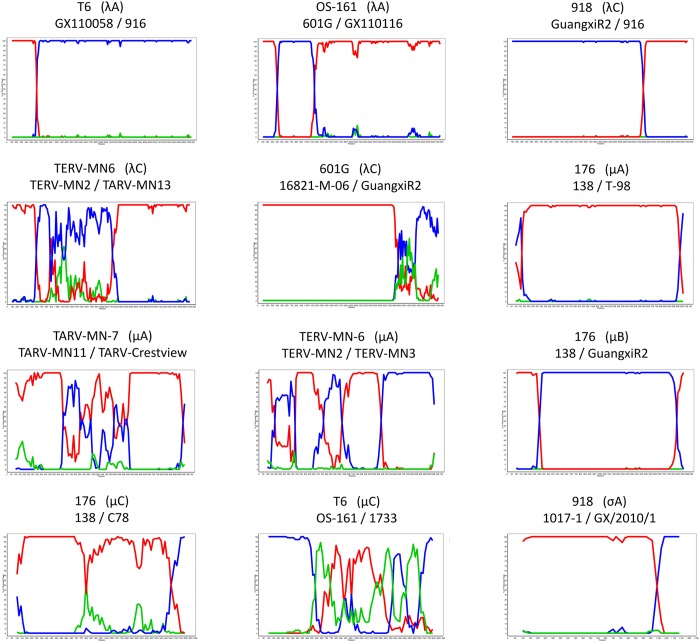
Bootscanning analysis of gallinaceous avian orthoreoviruses for detection of putative recombination events using the SimPlot program Version 3.5.1.

To uncover whether recombination involving cognate genome segments occurs in ARV, particularly the Hungarian isolates, we performed recombination analysis with the studied Hungarian strains and sequences of ARVs (chicken and turkey) available in GenBank. Analysis of the coding region of each genomic segment revealed twelve putative recombination events in the λA (2), λC (3), μA (3), μB (1), μNS (2), and σA (1) genes. Details of the analyses are provided in Table 4 and Fig. 4. Among the few identified cases, the Hungarian strains were involved in one possible recombination event, namely, the isolate 601 G was identified as a possible recombinant that may have arisen from a strain closely related to 16821-M-06 and another strain related to GuangxiR2. In the data set no recombination event was detected within the σC coding gene, suggesting that serotype diversity is not derived from this mechanism.

## Conclusions

Our results provide insight into the molecular characteristics of ARV strains randomly selected from a major strain collection. Genome sequencing and bioinformatics analyses of these strains provided new insight into the evolutionary mechanisms of ARV strains detected over a decade in Hungary. We conclude that lineage diversification, recombination involving cognate genome segments, reassortment among homologous strains and even among heterologous strains drive the evolution and genetic diversity of chicken orthoreovirus strains in Hungary. It remains to be studied whether similar mechanisms affect the genetic diversity of ARV strains in other parts of the world. A better understanding of the relative importance of these mechanisms will likely affect current vaccination strategies and the control and prevention of reovirus infections.

## Materials and Methods

### ARV isolates

Samples processed in this study originated from young chicken found dead following a course of disease with different manifestations in commercial poultry flocks between 2002 and 2011 in Hungary and submitted for post mortem evaluation to the Veterinary Diagnostic Directorate, National Food Chain Safety Office, Debrecen. Chicken ARV strains had been isolated from the collected specimens and the tissue culture supernatant of each isolate had been lyophilized for later use (Table 1). Prior to next generation sequencing, the lyophilized samples of ten randomly chosen strains had been dissolved in sterile double-distilled water. The routine diagnostic investigations were performed according to the current directives specified in law 1998./XXVIII. and 2008./XLVI., and regulation 41/1997. (V. 28.).

### Whole genome sequencing

Sequencing was performed using the protocol described in detail elsewhere^16^. In brief, RNA was extracted from diluted samples using TRI Reagent (Sigma-Aldrich, Saint Louis, MO, USA) with the Direct-zol RNA MiniPrep Kit (Zymo Research, Irvine, CA, USA) according to the manufacturer’s recommendations. Random primed reverse transcription was followed by amplification of complementary DNA (cDNA). A cDNA library was prepared using the NEBNext Fast DNA Fragmentation & Library Prep Set for Ion Torrent (New England Biolabs, Beverly, MA, USA) using the Ion Torrent Xpress Barcode Adapters (Thermo Fisher Scientific, Waltham, MA, USA). The emulsion PCR and subsequent templated bead enrichment was performed with a OneTouch v2 instrument and Ion OneTouch ES (Thermo Fisher Scientific), respectively. Sequencing was carried out on a 316 chip using the Ion Torrent Personal Genome Machine (Thermo Fisher Scientific). Sequences were assembled and aligned with the software CLC Genomics Workbench (http://www.clcbio.com). To determine the missing sequences of the coding regions specific primer sets were designed. Direct sequencing was carried out applying the BigDye Terminator v1.1 Cycle Sequencing Kit (Thermo Fisher Scientific) and run on an automated sequence analyzer (ABI PRISM 310 Genetic Analyzer, Applied Biosystems, Foster City, CA).

### Computer analyses

Contigs were aligned with Sanger sequencing reads using MultAlin online software^17^ and were edited in GeneDoc^18^. Phylogenetic analysis was performed with the MEGA6 program package^19^ based on multiple sequence alignments generated by the TranslatorX online platform^20^; the best-fit substitution models were selected for each gene-specific dataset based on the Bayesian information criterion. Maximum-likelihood trees were generated and tree topologies were validated by bootstrap analysis (100 replicates). Pairwise comparison of nucleotide sequences and amino acid p-distances of the Hungarian ARV strains and reference ARV strains, S1133, AVS-B and a Hungarian strain T1781 were calculated by applying the BioEdit^21^ and MEGA6 program package, respectively. The accession numbers of orthoreovirus strains used in sequence and phylogenetic analyses are listed in Supplementary Table S1. The aligned sequences of the Hungarian isolates and ARV sequences available from GenBank were subjected to recombination analysis. The Recombination Detection Program (RDP) package v.3.44^22^ was used for identification of recombinant sequences and breakpoints using the default parameters for the methods GENECONV, Bootscan, Chimaera, MaxChi, SiScan, 3Seq and RDP. Only those recombination events were taken into considerations that were supported by at least 4 methods to avoid misidentification using a single methodology. The best signals for recombination are associated with the lowest P-values; the highest acceptable P-value was set to 0.05. Recombination events detected with RDP v.3.44 were confirmed and visualized with SimPlot Version 3.5.1^23^. Analysis and visualization of aligned concatenated whole genome sequences was performed with mVISTA online platform^24^.

### GenBank accession numbers

The genome sequences of the Hungarian strains were deposited in GenBank under the following accession numbers: KX398232-KX398331.

No animal experiments were done in the study. The routine diagnostic investigations were performed according to the current directives specified in law 1998./XXVIII. and 2008./XLVI., and regulation 41/1997. (V. 28.).

## Additional Information

**How to cite this article**: Farkas, S. L. *et al*. Lineage diversification, homo- and heterologous reassortment and recombination shape the evolution of chicken orthoreoviruses. *Sci. Rep*. **6**, 36960; doi: 10.1038/srep36960 (2016).

**Publisher’s note:** Springer Nature remains neutral with regard to jurisdictional claims in published maps and institutional affiliations.

## Supplementary Material

Supplementary Information

## Figures and Tables

**Table 1:** The list of Hungarian chicken origin avian orthoreovirus strains randomly selected from a collection and processed in this study.

ARV isolate	Genbank accession number	Origin		Date of collection	Age of animals	Clinical signs, lesions
		*County*	*Settlement*			
*3211-V-02*	KX398272–KX398281	Hajdú-Bihar	Hajdúnánás	2002	No data	No data
*4599-V-04*	KX398292–KX398301	Szabolcs-Szatmár-Bereg	Kisvárda	2004	No data	No data
*875-Bi-05*	KX398242–KX398251	Borsod-Abaúj-Zemplén	Szakáld	2005	14 days	gastrointestinal signs, necrotic enteritis, airsacculitis, pericarditis, yolk sac retention
*878-Bi-05*	KX398252–KX398261	Hajdú-Bihar	Debrecen	2005	21 days	necrotic enteritis, heart failure
*924-Bi-05*	KX398262–KX398271	Borsod-Abaúj-Zemplén	Kesznyéten	2005	28 days	airsacculitis, pericarditis
*284-V-06*	KX398232–KX398241	Békés	Békéscsaba	2006	No data	uneven growth rate, gastrointestinal signs
*16821-M-06*	KX398302–KX398311	Szabolcs-Szatmár-Bereg	Ajak	2006	35 days	lameness, necrotic enteritis, pericarditis, respiratory signs
*17203-M-06*	KX398312–KX398321	Hajdú-Bihar	Görbeháza	2006	No data	airsacculitis, necrotic enteritis, pericarditis
*17227-M-10*	KX398322–KX398331	Hajdú-Bihar	Nagyhegyes	2010	14-28 days	necrotic enteritis, heart failure, nephritis, pericarditis
*3457-M-11*	KX398282–KX398291	No data	No data	2011	37 days	uneven growth rate, necrotic enteritis, gout, foot pad ulcer

**Table 2:** Gene-specific nucleotide (nt) and amino acid (aa) sequence identities (%) and similarities (%) of the studied Hungarian avian orthoreoviruses to other avian origin reoviruses (Muscovy duck reovirus strains: J18, D2044, D1546; Turkey reovirus strains: TARV-MN2, D1104, D1246, 19831 M; Partridge reovirus: D1007).

Strain	λA	λB	λC	μA	μB	μNS	σC	σA	σB	σNS
NT										
Hungarian chicken	81.5–98.8	78.8–99.8	71.1–99.8	77.5–99.7	**62.7**–**99.8**	79.4–99.9	**52.9**–**100.0**	80.9–99.8	83.1–99.7	78.8–100.0
T1781	81.7–95.1	79.9–90.3	71.1–93.9	80.3–86.2	**65.3**–**93.7**	79.9–98.0	**43.8**–**58.5**	81.4–98.0	83.1–98.6	80.6–99.3
AVS–B	82.5–93.3	81.5–93.8	73.2–90.6	77.6–95.8	**64.0**–**76.7**	79.4–92.7	**54.9**–**69.1**	80.4–89.6	83.6–90.1	79.6–98.8
S1133	83.1–89.7	80.2–87.2	70.5–82.0	77.3–88.6	**64.0**–**85.1**	79.9–87.0	**54.3**–**78.8**	80.7–90.7	85.3–91.0	78.7–83.2
D1007	78.0–79.0	79.4–84.5	70.6–84.8	80.2–86.4	**63.9**–**78.6**	80.1–87.0	**52.2**–**59.5**	81.0–88.0	81.6–84.4	80.7–82.6
Turkey	81.6–84.0	78.4–83.5	78.4–83.5	77.2–86.3	**64.4**–**77.1**	78.8–86.4	**48.8**–**60.4**	76.0–89.2	58.7–72.1	73.0–80.9
Muscovy duck	76.3–78.8	72.0–76.6	72.0–76.6	70.4–74.4	**60.5**–**77.3**	70.7–72.3	**26.8**–**39.6**	75.6–78.0	60.7–65.9	78.5–81.2
**AA**										
Hungarian chicken	96.5–99.0	96.0–100.0	83.0–100.0	92.9–99.9	**71.3**–**99.7**	90.1–100.0	**46.2**–**100.0**	96.7–100.0	94.2–100.0	90.1–100.0
T1781	97.0–99.3	96.7–98.3	83.3–98.2	93.6–97.3	**72.6**–**98.6**	91.2–98.9	**51.4**–**72.4**	96.9–99.7	94.2–99.4	90.7–99.4
AVS–B	97.0–99.1	95.9–99.1	83.7–96.8	93.6–96.6	**71.1**–**90.2**	90.2–96.7	**50.5**–**75.5**	96.4–97.9	95.5–97.1	90.7–100.0
S1133	97.0–98.0	95.9–96.9	83.7–93.8	93.4–98.7	**72.5**–**95.0**	91.3–93.8	**46.7**–**81.9**	95.6–96.7	95.5–97.7	92.1–94.2
D1007	95.1–95.6	94.8–96.0	81.7–91.8	92.2–95.8	**72.4**–**94.2**	89.3–95.6	**49.5**–**55.2**	96.4–98.7	87.7–90.6	91.8–93.0
Turkey	96.2–97.1	94.5–95.9	81.1–90.9	91.7–95.4	**71.3**–**90.2**	88.8–92.1	**50.0**–**55.7**	95.4–97.2	77.1–80.3	90.1–92.1
Muscovy duck	93.6–95.4	90.4–91.2	77.7–79.5	84.2–86.6	**68.9**–**90.8**	79.5–80.9	**27.1**–**30.5**	91.0–92.6	61.9–70.6	89.8–92.1

**Table 3:** 

		NT															
		924-Bi-05	3457-M-11	16821-M-06	S1133	AVS-B	T1781	MRV-1	MRV-2	MRV-3	BroV	BRV	47/02	NBV	TVAV	Pycno-1	SSRV
AA	924-Bi-05	ID	95.3	98.0	66.2	65.6	65.3	47.5	45.5	46.3	49.5	45.6	52.8	61.6	64.4	64.5	64.0
	3457-M-11	99.7	ID	95.5	63.9	63.9	64.3	46.3	44.1	45.6	47.8	44.3	51.6	59.6	62.3	62.6	62.5
	16821-M-06	99.4	99.4	ID	65.8	65.8	65.1	47.2	45.3	46.4	49.3	46.1	52.9	61.2	64.5	64.6	64.1
	S1133	72.6	72.8	72.5	ID	75.7	73.3	47.5	46.6	46.9	49.4	47.4	53.8	63.6	65.7	66.7	68.0
	AVS-B	72.2	71.9	71.7	89.0	ID	71.9	47.7	46.7	46.7	50.8	47.2	54.3	63.6	65.7	66.7	68.0
	T1781	73.4	73.3	73.1	85.7	88.5	ID	47.6	47.1	47.1	52.2	45.6	54.5	62.8	66.9	67.1	67.2
	MRV-1	44.8	44.8	44.8	45.1	45.1	46.3	ID	74.7	82.9	44.9	42.0	47.0	46.7	47.5	47.9	48.4
	MRV-2	44.9	44.9	44.9	45.6	45.7	46.8	97.8	ID	76.5	42.8	41.3	46.1	45.5	47.7	47.7	47.7
	MRV-3	45.3	45.3	45.3	45.3	45.1	46.8	98.0	97.7	ID	44.1	41.0	45.8	45.4	46.6	47.8	47.3
	BroV	45.7	45.7	45.6	47.4	47.4	48.2	40.9	40.6	41.1	ID	47.6	53.9	51.1	50.8	50.8	50.5
	BRV	39.7	39.8	39.5	39.3	40.0	39.5	34.5	34.4	34.5	37.9	ID	50.2	46.5	47.4	47.7	47.1
	47/02	51.0	50.9	50.9	51.2	52.9	53.2	42.6	42.8	42.8	49.0	40.1	ID	53.4	54.8	54.9	54.4
	NBV	64.5	64.4	64.4	68.1	69.2	69.7	44.3	44.6	44.3	46.5	40.7	52.7	ID	65.5	65.6	66.1
	TVAV	68.6	68.4	68.3	72.9	75.6	73.7	45.1	45.4	44.9	46.8	42.0	54.7	72.6	ID	70.8	71.9
	Pycno-1	69.1	68.9	68.3	77.4	79.0	77.9	47.0	47.4	46.7	48.4	42.0	54.0	74.0	84.9	ID	75.8
	SSRV	68.4	68.6	68.4	76.4	78.2	77.3	47.3	47.7	47.0	47.9	41.5	53.2	73.9	83.2	93.2	ID

Nucleotide (nt) and amino acid (aa) sequence identities (%) and similarities (%) of the studied Hungarian avian orthoreoviruses (924-Bi-05, 3457-M-11, 16821-M-06) to representative strains of the five established *Orthoreovirus* species (*Mammalian orthoreovirus*, MRV: MRV-1 strain Lang, MRV-2 strain Jones, MRV-3 strain Dearing; *Avian orthoreovirus*: Avian orthoreovirus strain S1133; *Nelson Bay orthoreovirus*, NBV: Nelson Bay virus; *Reptilian orthoreovirus*: Bush viper reovirus strain 47/02; *Baboon orthoreovirus*: BRV: Baboon orthoreovirus), respectively, and strains, *Broome virus* (BRV), Steller sea lion reovirus (SSRV), Bulbul orthoreovirus (Pycno-1), and Tvärminne avian virus (TVAV) belonging to putative *Orthoreovirus* species.

**Table 4:** Results of the recombination analyses performed with the aligned sequences of avian orthoreoviruses of gallinaceous birds available from GenBank applying recombination detection program RDP v.3.44.

Segment (gene)	Recombinant strain	Parent major /minor	Regions derived from minor parent	Model							Recombinant score	Possibility (MC corrected)
				RDP Av. P-Val	GENECONV Av. P-Val	BootScan Av. P-Val	MaxChi Av. P-Val	Chimera Av. P-Val	SiScan Av. P-Val	3Seq Val		
L1 (λA)	T6	GX110058/916	1–566 3858–3882	—	1,598 × 10^−42^	5,005 × 10^−45^	1,772 × 10^−15^	1,767 × 10^−15^	2,011 × 10^−16^	9,294 × 10^−56^	0.685	9,294 E-56
	OS161	601 G/GX110116	444–1162	3,075 × 10^−36^	7,945 × 10^−39^	1,766 × 10^−38^	6,930 × 10^−10^	9,081 × 10^−15^	9,528 × 10^−24^	1,766 × 10^−19^	0.744	Global KA P-value 7,945 E-39
L2 (λC)	918	GuangxiR2/916	2816–3830	2,209 × 10^−93^	6,072 × 10^−88^	1,698 × 10^−93^	3,520 × 10^−30^	1,704 × 10^−30^	4,473 × 10^−38^	2,768 × 10^−131^	0.758	2,768 E-131
	TERV-MN6	TERV-MN2/TARV-MN13	498–2186	1,040 × 10^−10^	5,952 × 10^−04^	1,158 × 10^−09^	3,224 × 10^−16^	2,885 × 10^−18^	3,279 × 10^−13^	4,456 × 10^−32^	0.749	4,456 E-32
	601 G	16821-M-06/GuangxiR2	1–2820 3836–3859	5,725 × 10^−35^	1,850 × 10^−29^	3,234 × 10^−46^	1,380 × 10^−25^	3,144 × 10^−15^	3,080 × 10^−64^	1,323 × 10^−11^	0.602	3,234 E-46
M1 (μA)	176	138/T-98	260–1998	—	—	5,043 × 10^−06^	3,015 × 10^−05^	1,524 × 10^−05^	2,966 × 10^−32^	8,862 × 10^−20^	0.666	8,862 E-20
	TERV-MN6	TERV-MN2/TERV-MN3	1498–2152	6,493 × 10^−08^	2,853 × 10^−10^	2,159 × 10^−13^	4,322 × 10^-08^	3,671 × 10^-08^	8,321 × 10^−12^	3,855 × 10^−11^	0.694	Binomial probability 2,159 E-13
	TARV-MN7	TARV-MN11/TARV-Crestview	1–700 1358–1965	—	—	1,029 × 10^−03^	2,714 × 10^−05^	2,583 × 10^-05^	1,945 × 10^−11^	—	0.711	Region probability 2,583 E-05
M2 (μB)	176	138/GuangxiR2	286–1825	2,327 × 10^−14^	5,954 × 10^−12^	9,800 × 10^−13^	8,389 × 10^−14^	9,913 × 10^−14^	1,709 × 10^−37^	3,861 × 10^−46^	0.744	3,861 E-46
M3 (μNS)	176	138/C-78	1–1691 1877–1909	—	—	—	6,570 × 10^−03^	5,519 × 10^−03^	1,536 × 10^−25^	1,226 × 10^−11^	0.513	Possible misidentification of recombinant
	T6	OS161/1733	1–568 1636–1909	5,611 × 10^-07^	1,227 × 10^−04^	2,679 × 10^−09^	8,611 × 10^−09^	1,345 × 10^−06^	2,024 × 10^−08^	—	0.673	2,679 E-09
S2 (σA)	918	1017-1/GX/2010/1	1–1023 1222–1252	4,427 × 10^−03^	—	4,471 × 10^−03^	6,956 × 10^-06^	3,864 × 10^−04^	1,206 × 10^−25^	4,603 × 10^−14^	0.749	4,603 E-14

Putative recombination events supported by at least four methods were listed.
