# Supplementary material for: Lineage diversification, homo- and heterologous reassortment and recombination shape the evolution of chicken orthoreoviruses
Source: Sci Rep. 2016 Nov 10;6:36960. doi: 10.1038/srep36960 (PMC5103266; doi:10.1038/srep36960)
Supplement: Supplementary Information [file srep36960-s1.pdf]

Lineage diversification, homo- and heterologous reassortment and recombination shape the evolution of chicken orthoreoviruses

Szilvia L. Farkas[1,✉], Szilvia Marton[1], Eszter Dandár[2], Renáta Kugler[1], Bence Gál[1], Ferenc Jakab[3], Ádám Bálint[4], Sándor Kecskeméti[5], Krisztián Bányai[1]

[1]Institute for Veterinary Medical Research, Centre of Agricultural Research, Hungarian Academy of Sciences, Hungária krt. 21, Budapest 1143, Hungary

[2]United Szent István és Szent László Hospital – Clinic, Nagyvárad tér 1, Budapest 1097, Hungary

[3]János Szentágothai Research Centre, University of Pécs, Ifjúság útja 20, Pécs 7624, Hungary

[4]Veterinary Diagnostic Directorate, National Food Chain Safety Office, Tábornok u. 2, Budapest 1143, Hungary

[5]Veterinary Diagnostic Directorate, National Food Chain Safety Office, Bornemissza u. 3-7, Debrecen 4031, Hungary

✉Corresponding author

E-mail: fszilvi@yahoo.com

Oszlop1	HB10-1	LN09-1	SD09-1	SD10-1	GX/2010/1	GX110116
L1	KP288827	KP288837	KP288847	KP288857	KJ476699	KF741746
L2	KP288828	KP288838	KP288848	KP288858	KJ476700	KF741747
L3	KP288829	KP288839	KP288849	KP288859	KJ476701	KF741748
M1	KP288830	KP288840	KP288850	KP288860	KJ476702	KF741749
M2	KP288831	KP288841	KP288851	KP288861	KJ476703	KF741750
M3	KP288832	KP288842	KP288852	KP288862	KJ476704	KF741751
S1	KP288833	KP288843	KP288853	KP288863	KJ476705	KF741752
S2	KP288834	KP288844	KP288854	KP288864	KJ476706	KF741753
S3	KP288835	KP288845	KP288855	KP288865	KJ476707	KF741754
S4	KP288836	KP288846	KP288856	KP288866	KJ476708	KF741755

C78	C-98	GuangxiR1	1733	S1133	GuagnxiR2	GX110058	176
KF741716	EU616735	KC183748	KF741706	KF741756	KF741726	KF741736	EU707934
KF741717	JN641888	KC183749	KF741707	KF741757	KF741727	KF741737	EU707936
KF741718	EU616737	KC183750	KF741708	KF741758	KF741728	KF741738	EU707938
KF741719	EU616740	KC183751	KF741709	KF741759	KF741729	KF741739	AY557189
KF741720	EU616741	KC183752	KF741710	KF741760	KF741730	KF741740	AY750053
KF741721	EU616744	KC183743	KF741711	KF741761	KF741731	KF741741	AY557191
KF741722	EF057397	KC183744	KF741712	KF741762	KF741732	KF741742	AF218358
KF741723	JN641886.	KC183745	KF741713	KF741763	KF741733	KF741743	AF059716
KF741724	EF030496	KC183746	KF741714	KF741764	KF741734	KF741744	AF059720
KF741725	JN641885	KC183747	KF741715	KF741765	KF741735	KF741745	AF059724

138	2408	919	T-98	T6	R2/TW	1017-1	916
EU707933	AY641742	AY641739	EU616739	DQ238094	AY641744	AY641740	AY641737
EU707935	0	0	JN641889	0			
EU707937	AY652694	AY652697	EU616738	AY652698	DQ238095	DQ238096	AY652701
AY557190	AY639613	AY639618	EU616736	AY639621	AY639620	AY639611	AY639616
AY750052	AY635937	AY635939	EU616742	AY635936	AY635940	AY635935	AY635943
AY557190	AY573907	AY573912	EU616743	AY573915	AY573914	AY573905	AY573910
AF218359	AF204945	AY573912	EF057398	AF204948	AF297213	AF297216	AF297214
AF059717	AF247724	AF294763	JN641887	AF294768	AF294765	AF294762	AF294764
AF059721	AF208038	AF208034	EF030499	AF208036	AF301472	AF301474	AY008383
AF059725	AF213468	AF294776	JN641884	AF213469	AF294778	AF294771	AF294774

918	Reo/PA/Broiler/15511/13	Reo/PA/Broiler/05682/12	AVS-B	OS-161
AY641738	KP731611	KM877325	FR694191	AY641743
	KP731612	KM877326	FR694192	
AY652700	KP731613	KM877327	FR694193	AY652696
AY639617	KP731614	KM877328	FR694194	AY639619
AY635945	KP731615	KM877329	FR694195	AY635944
AY573911	KP731616	KM877330	FR694196	AY573913
AF297215	KP731617	KM877331	FR694197	AF204946
AF294766	KP731618	KM877332	FR694198	AF294770
AF301473	KP731619	KM877333	FR694199	AF301471
AF294775	KP731620	KM877334	FR694200	AF294777

750505	19831M	D1246	D1104	Turkey/USA/MN/2011/TARV-MN2	D1007
DQ238093	KR997899	KR997909	KR997919	KJ865903	KR476798
	KR997900	KR997910	KR997920	KJ865893	KR476799
AY652695	KR997901	KR997911	KR997921	KJ865883	KR476800
AY639615	KR997902	KR997912	KR997922	KJ874307	KR476801
AY635942	KR997903	KR997913	KR997923	KJ874284	KR476802
AY573909	KR997904	KR997914	KR997924	KJ874261	KR476803
AF204950	KR997905	KR997915	KR997925	KF872233	KR476804
AF294767	KR997906	KR997916	KR997926	KF872246	KR476805
AF208035	KR997907	KR997917	KR997927	KF872255	KR476806
AF213470	KR997908	KR997918	KR997928	KF872270	KR476807

J18	D20/99	D1546	D2044	Bush viper reovirus	NBV	SSRV
JX478260	KF809662	KJ871017	KJ871007			
JX478261	KF809663	KJ871019	KJ871009			
JX478262	KF809664	KJ871018	KJ871008			
JX478263	KF809665	KJ871020	KJ871010			
JX478264	KF809666	KJ871021	KJ871011	NC_023820	JF342676	HM222976
JX478265	KF809667	KJ871022	KJ871012			
JX478266	KF809668	KJ871023	KJ871013			
JX478267	KF809669	KJ871024	KJ871014			
JX478268	KF809670	KJ871025	KJ871015			
X478269	KF809671	KJ871026	KJ871016			

Tvarminne avian reovirus	Tortoise reovirus CH1197/96	T1781	Pycno-1
KF692093	KT696551	KC865790	AB914764

Broome virus	Baboon orthoreovirus	MRV Lang	MRV Dearing	MRV Jones
NC_014240	NC_015881	AF490617	EF494439	M19355
